# Supplementary material for: Impact of self-administered pulse oximetry among non-hospitalized patients at risk of severe COVID-19 in Honduras: A pragmatic, cluster-randomized trial with temporal clustering
Source: PLOS Glob Public Health. 2025 Nov 7;5(11):e0004618. doi: 10.1371/journal.pgph.0004618 (PMC12594330; doi:10.1371/journal.pgph.0004618)
Supplement: S1 Protocol — (PDF) [file pgph.0004618.s002.pdf]

Implementation and Evaluation of Home-based Care and Hand Hygiene Interventions in Honduras

Detailed Protocol – Mass General Brigham IRB

Honduras Secretaría de Salud (SESAL)

Brigham and Women's Hospital (BWH)

Centers for Diseases Control and Prevention (CDC)

5 May 2022
Version 5.1

Contents

List of Acronyms ... 3

Project Summary ... 4

Investigators and Institutional Affiliations ... 4

Funding Source ... 5

Background ... 6

Goal, Objectives, and Activities ... 7

- Goal ... 7
- Objectives ... 7
- Activities ... 7

Project overview ... 8

- Rationale ... 8
- Setting ... 8
- Project Population ... 8
- Inclusion and Exclusion Criteria ... 8
- Recruitment procedures ... 9
- Home-based care strategies ... 9
- Referral to higher level of care ... 9
- Home-based care kits and strategies ... 9
- Discharge from daily clinical monitoring ... 9
- Data collection and survey tools ... 10

Indicators ... 12

Data Management ... 12

- Data collection instruments ... 12
- Data monitoring dashboard ... 12

Sample size for primary outcome indicators ... 13

Data analysis ... 14

- Training and Qualifications of Personnel ... 14

Risks and Benefits to Participants ... 14

Human subjects' considerations ... 15

Data sharing and dissemination policies ... 15

References ... 17

List of Acronyms

ABHS	Alcohol-Based Hand Sanitizer
BWH	Brigham and Women's Hospital
CDC	the Centers for Disease Control and Prevention
CETTE	Centros Temporales de Triaje y Estabilización
DGRISS	Dirección General de Redes Integradas de Servicios de Salud
MOH	Ministry of Health
SESAL	Secretaría de Salud
WASH	Water, sanitation, and hygiene
WHO	World Health Organization

Project Summary

This protocol describes an evaluation of home-based care (HBC) and hand hygiene practices for COVID-19 patients and their families in Honduras. The World Health Organization (WHO) states that "Home care may be considered for an adult or child with confirmed or suspected COVID-19 when inpatient care is unavailable or unsafe… [or for] patients who have been discharged from hospital."[1] In Honduras, the Secretaría de Salud (SESAL) has published guidelines for home-based care for patients with confirmed or suspected COVID-19 infection, but there is currently no formal mechanism for monitoring HBC.[2] Evaluation of the use of pulse oximetry to screen COVID-19 patients for hypoxia is needed to determine whether this practice leads to improved patient outcomes for COVID-19 patients receiving HBC. The current project aims to generate operationally relevant data that can be used to guide decisions regarding HBC strategies, and evaluate the feasibility and impact of pulse oximetry for patients receiving HBC. Nested within the HBC study, we will also implement and evaluate a hand hygiene intervention among the project participants, examine intervention acceptability among clinical staff, and assess long-term COVID-19 symptoms. While most persons with COVID-19 recover and return to normal health, some patients can have symptoms that can last for weeks or even months after recovery from acute illness. Given the incidence and changes over time of these symptoms, we will monitor and assess long term symptoms among COVID-19 patients.

Ethical Review Boards

The study PI is responsible for ensuring that all ethical approval requirements have been met and ethical approval will be obtained from Mass General Brigham IRB and the National Autonomous University of Honduras Faculty of Medical Sciences Bioethics Committee (Comité de Ética en Investigación Biomédica, CEIB).

Investigators and Institutional Affiliations

CDC

CDC staff will provide technical support. CDC staff will not collect data or interact with project or study participants. CDC is not engaged in this activity.

Brigham and Women's Hospital

- Eric J. Nilles, MD, MSc, PI, will be responsible for overall programmatic and administrative direction and outcomes, including study design, IRB approval, ensuring human subject protection, and data collection, analysis, interpretation, and dissemination of results. Phone +1-781-995-7077, email enilles@bwh.harvard.edu.
- Dan Schnorr, MD, MPH, Co-Investigator, responsible for protocol development, study planning, implementation, data analysis, reporting, and dissemination of results. Phone: +1-978-376-3201, Email: cschnorr@bwh.harvard.edu
- Berta Alvarez, MPH, Co-investigator and Study Coordinator, in coordination with Honduras health authorities will be responsible for study implementation, in-country day to day operations, monitoring enrollment of patients and adherence to study protocols, and study reporting, and dissemination of results.
- Kathryn Roberts, MPH, Co-Investigator and study epidemiologist. Responsible for protocol development, study planning, implementation, oversight, data analysis, reporting, and dissemination of results. Phone: +1-415-215-2710 Email: kroberts@bwh.harvard.edu

Secretaria de Salud (SESAL) of Honduras

- Alcides Martínez, MD, MPH, will be the focal point for this study within the service network of SESAL, responsible for keeping national health authorities informed, generating internal and external coordination mechanisms, and making study decisions based on evidence and the objectives defined in the protocol.
- Jonatán Ochoa, MD, will be the focal point for this study within DGRISS, responsible for coordination between DGRISS and remainder of the study team and for assuring that study procedures are aligned with SESAL regulations and protocols.
- Yisela Martínez NR, MSc. will be the focal point for this study within the health services network, responsible for coordination between healthcare facilities within the health region, including primary care centers, Triage Centers (CETTE) and higher levels of care.
- Karla Rosales Pavón, MD, MSc. Chief of SESAL Surveillance Unit, will serve as focal point for communication with in-country regional epidemiologists, assist with collection and analysis of national COVID-19 data and selection of triage centers for study sights. Will also support analysis and dissemination of study data.
- Homer Mauricio Mejía Santos. MD, MSc. coordinator for communicable and non-communicable diseases for SESAL Surveillance Unit, will assist with collection and analysis of national COVID-19 data and selection of triage centers for study sights. Will also support analysis and dissemination of study data.
- Lorenzo Pavón, MD, PhD. Head of the SESAL Surveillance Unit (from 2022), will serve as a focal point for communication with regional epidemiologists in the country, will assist with the collection and analysis of national COVID-19 data. He will also support the analysis and dissemination of the study data.

US Centers for Disease Control and Prevention, Central America

- Emily Zielinski-Gutierrez, MPH, DrPH, Co-Investigator. Responsible for design, monitoring of implementation, analysis, reporting, and dissemination of results. ebz0@cdc.gov, +502 55997732
- Nasim Farach, MPH, Co-Investigator. Responsible for design, monitoring of implementation, analysis, reporting, and dissemination of results.

US Centers for Disease Control and Prevention, Atlanta

- Avi Hakim, MA, MPH, Co- Investigator. Responsible for study design, protocol development, monitoring, analysis, interpretation, and dissemination of results. Phone: +1-404-374-4686, email: hxv8@cdc.gov
- Matthew Lozier, PhD, MPH, Co-Investigator. Responsible for design, monitoring of implementation, analysis, reporting, and dissemination of results. mlozier@cdc.gov 404-718-7797.

Funding Source

This evaluation is funded by the U.S. Centers for Disease Control and Prevention (CDC) under the terms of the cooperative agreement U01GH002238, Reducing the morbidity and mortality due to acute febrile illnesses in Central America and the Dominican Republic.

Background

As of December 16, 2021, there have been more than 271 million confirmed cases of COVID-19 and 5.3 million deaths worldwide.[3] Secretary General of the United Nations Antonio Guterres has stated that the impact of the COVID-19 pandemic has fallen "disproportionately on the most vulnerable: people living in poverty, the working poor, women and children, persons with disabilities, and other marginalized groups."[4] In Honduras, as of April 12th, 2021, there had been 378,642 confirmed cases of COVID-19, leading to 10,424 deaths.[5]

While most COVID-19 patients have mild or moderate symptoms, some individuals develop serious or critical signs and symptoms.[3] Persons with marked hypoxia may have normal respiratory rates but later develop severe symptoms or respiratory failure.[6–8] Timely detection and transfer of persons with hypoxia or other alarm signs and symptoms to a higher level of care is critical.

According to the WHO, "home pulse oximetry is a safe, non-invasive way to assess oxygen saturation in the blood and can support the early identification of low oxygen levels in a patient with initially mild or moderate COVID-19 or silent hypoxia, when a patient does not appear to be short of breath but his or her oxygen levels are lower than expected."[1] Pulse oximetry can also identify patients for whom it is safe to remain in HBC, thereby reducing unnecessary use of advanced care resources, which are frequently limited.[9] There is currently limited evidence on the impact, feasibility, and acceptability of home-based pulse oximetry in resource-limited settings.

The Honduras SESAL has established an extensive network of COVID-19 triage centers to identify, test and triage potential COVID-19 patients. These centers are known by their Spanish acronym CETTE (Centros Temporales de Triaje y Estabilización). Patients that do not require hospitalization are discharged to home. However, there have been difficulties with following and monitoring these patients and at present no mechanism monitors outcomes for these patients. Therefore, this project proposes to establish a framework to provide structured HBC for high-risk patients to include symptom and clinical monitoring, and guidance on when and how to seek additional medical care. Further, at present, the utility of pulse oximetry for monitoring of COVID-19 home-based care patients in lower- and middle-income settings has not been established and although potentially beneficial is not the standard of care in most countries including the US.[10,11]

Practicing hand hygiene frequently with appropriate technology and technique can mitigate the spread of COVID-19. The standard of care is handwashing with soap and water, scrubbing hands for at least 20 seconds. The use of alcohol-based hand sanitizer (ABHS) with at least 60% alcohol content is another hand hygiene method that can mitigate COVID-19.[12] Knowledge about appropriate technique and situations to use each method varies, as does personal preference. However, there is a knowledge gap about whether provision of hand hygiene resources leads to increasing reported hand hygiene practices, especially in low- and middle-income countries, and this setting is key to mitigating household transmission of COVID-19.

Goal, Objectives, and Activities

Goal

To support the SESAL of Honduras to mitigate the impact of the COVID-19 pandemic by reducing morbidity and mortality among COVID-19 patients managed through existing home-based care strategies with signs and/or diminished blood oxygen level, and by preventing the spread of SARS-CoV-2 to household contacts of patients being managed in this setting.

Objectives

1) To support and strengthen existing HBC for higher risk COVID-19 patients in Honduras
2) To generate operationally relevant data to provide the Honduras SESAL and other health authorities with HBC guidance and protocols.
3) To assess the utility and impact of pulse oximetry on outcomes for HBC.
4) To evaluate differences in resource utilization by HBC strategy.
5) To assess acceptability of different HBC strategies by patients, study staff, and SESAL clinicians staffing triage centers.
6) To assess the prevalence of post COVID-19 conditions.
7) To assess participant-reported in-home hand hygiene practices before and after receiving HBC kit.
8) To assess if participants with access to alcohol-based hand sanitizer (ABHS) practice hand hygiene more frequently.
9) To evaluate preferences for different hand hygiene technologies.

Activities

1) Screening of suspected COVID-19 patients
2) Confirmation of COVID-19 using rapid SARS-CoV-2 Antigen testing.
3) Enrollment of eligible patients into one of the two HBC strategies.
4) Administration of enrollment questionnaire to collect data on demographics, clinical variables, medical history, risk factors, and patient-reported in-home hand hygiene practices.
5) Provision of COVID-19 HBC kits to all study participants, with and without alcohol-based hand sanitizer.
6) Daily phone-based or clinical monitoring of HBC patients, with referral of patients reporting warning signs, according to the national guidelines, to a higher level of care.
7) Chart review of study participants referred to a higher level of care to assess clinical features and outcomes.
8) Administration of serial phone-based questionnaires to study participants, including at discharge from HBC daily monitoring, to assess acceptability of HBC strategy, participant-reported in-home hand hygiene practices, and other WASH and socio-behavioral parameters.
9) Administration of questionnaires to study staff, triage center staff, and other relevant stakeholders to assess acceptability of various HBC strategies.
10) Administration of qualitative interviews to a small subset of patients to understand their hand hygiene practices, with and without ABHS.
11) Longitudinal serial phone-based questionnaires to assess incidence and temporal features of COVID-19 compatible symptoms among study participants, with referral for medical evaluation as needed.

Project overview

Rationale

This project is intended to enhance the existing SESAL HBC approach by providing additional resources and monitoring for patients with confirmed COVID-19 that are discharged home after diagnosis. In addition, given limited data on which home-based care strategies most effectively and efficiently minimize poor outcomes, we aim to monitor two discrete home-based care approaches, daily phone calls from nurses to assess the patient's clinical status, with and without the use of pulse oximeters by patients at home. Assignment to home-based care approach will be randomized. Given the primary goal of the project is to reduce morbidity and mortality due to the COVID-19 pandemic, we will not include a control arm.

Setting

The project location will be in existing SESAL triage centers, and other COVID screening locations, in the Municipalities of the Central District of Honduras (Tegucigalpa and Comayaguela). Selection of target **COVID screening locations** will be performed in collaboration with the Honduras SESAL and based on criteria including current and projected COVID-19 hotspots, mortality data and logistical considerations including access and security. Depending on patient enrollment and other considerations, other health facility types and locations may be included for enrollment.

Project Population

The study population is patients ≥ 45 years old with confirmed COVID-19 assessed to be higher-risk for poor outcomes but clinically stable and suitable for home-based care according to existing Honduras SESAL COVID-19 protocols. The assessment of which patients will be discharged to home will be conducted by SESAL clinicians according to existing SESAL protocols.

Inclusion and Exclusion Criteria

Inclusion criteria:

- Acute infection with SARS-CoV-2 confirmed by rapid antigen test.
- Patients triaged to HBC by SESAL clinicians according to existing SESAL policies and protocols.
- **45-59 years of age with at least one comorbidity related to increased risk for poor outcomes due to COVID-19 or 60+ years of age.**
- Live or work in the city where the study is being implemented
- Agrees to participate and signs informed consent

Exclusion criteria:

- Negative for SARS-CoV-2 by rapid antigen test
- Patients not triaged to HBC by SESAL clinicians
- Less than 45 years of age, or 45 - 59 years without any comorbid conditions that increase the risk for poor outcomes due to COVID-19
- Does not live or work in the city where the study is being implemented
- Difficulty giving consent to participate due to a medical condition at the time of enrollment

Patients that do not meet all the inclusion criteria above will be excluded from project enrollment.

Recruitment procedures

Screening and recruitment will occur at the COVID screening locations. Patients that are evaluated by SESAL clinicians and triaged to HBC will undergo further screening by project staff to assess if they meet project inclusion criteria. Those that (i) are triaged to HBC by SESAL clinicians and (ii) meet the additional project inclusion criteria will be tested for SARS-CoV-2 via rapid antigen test. Those that receive positive test results will be offered enrollment by study staff.

Home-based care strategies

At the time of enrollment, participants will be assigned to one of the two following home-based care strategies using a block randomization approach, based on day of presentation to the triage center:

- Strategy 1: Study staff will perform daily phone-based assessments using standardized electronic study tools (tablets). Clinical features will be documented on a phone-based patient monitoring form.
- Strategy 2: Study staff will perform daily phone-based assessments using standardized electronic study tools (tablets). Clinical features and oxygen saturation levels will be documented on a phone-based patient monitoring form. Patients will be provided with a portable pulse oximeter for home use ("MIGHTYSAT" manufactured by MASIMO) or other device certified by the US Food and Drug Administration or European CE). Oxygen saturation level will be assessed up to four times per day (morning, midday, evening, plus during the phone-based assessment) by the patient and/or family member and the lowest stable (i.e. without substantial fluctuations) level recorded during a monitoring period of one minute will be recorded and registered. Patients will be provided monitoring sheets to record the times and dates of the oxygen saturation levels.

Study staff performing patient evaluations and monitoring will be doctors or nurses.

Referral to higher level of care

Patients meeting referral criteria of $SpO_2<92\%$ or other alarm signs will be referred to the nearest COVID triage center or hospital for care by study staff according to existing SESAL guidelines.

Home-based care kits and strategies

All study participants will receive COVID-19 HBC kits that include soap, face coverings, information sheets on COVID-19 prevention, and other supplies. Half the COVID-19 home kits will include a two-week supply of ABHS. Study participants will be block randomized into ABHS or no ABHS groups according to the day of the week they are tested for COVID-19. Patients assigned to Strategy 2 will be supplied with a portable pulse oximeter and trained on use and measurement by study staff prior to discharge from the triage center. All patients will be asked to return the portable pulse-oximeter when they are discharged from the study. Upon return of the portable pulse-oximeter at the end of the monitoring period, these patients may receive a phone card or similar item of approximately $10 value to help defray the costs of transportation. Patients who are triaged to HBC but are deemed ineligible for study enrollment may also be provided with an HBC kit if resources are available.

Discharge from daily clinical monitoring

All patients will receive daily monitoring until they are discharged from the daily monitoring program.

Criteria for discharge are:

1. Ten days post-symptoms onset and 24 hours without fever. Patients meeting these criteria will be discharged from the daily monitoring program. If a patient continues to experience fever more than 10-days post-symptoms onset they will continue to receive daily monitoring until they are fever-free for more than 24 hours.

OR

2. Patient is hospitalized. Patients that are not hospitalized will continue in the daily monitoring program until criterion #1 is met.

Data collection and survey tools

1. Enrollment questionnaire

All enrolled patients will complete an enrollment questionnaire that will include demographic and clinical information, risk factors, hand hygiene practices and other WASH variables, and socio-behavioral questions.

Questions will include timing and nature of symptoms, and COVID-19 compatible symptoms, if anyone else in the household has had COVID-19 symptoms in the past 30 days, and if so, when they began and how long they lasted. In order to later link patients with COVID testing results and follow-up visits to the CETTE or hospital in a SESAL database, we will also collect the patient's national identification number, but a unique study identification number will also be assigned. The enrollment questionnaire will also assess risk factors of the participant and household members such as which household members leave the house for work or school and if there are any other possible exposures to COVID-19. Specific questions about the WASH component include assessing the participants' knowledge, attitudes, and practices about hand hygiene in the month prior to visiting the triage center. The enrollment questionnaire will assess access to hand hygiene resources in the participants' homes. The estimated time to complete enrollment and questionnaire is 20-25 minutes.

2. Daily acute COVID-19 symptom monitoring

Study staff will document clinical features, including oxygen saturation when applicable, and alarm signs or symptoms during each daily follow-up phone call using a standardized form. Data on COVID-19 compatible symptoms among household members will also be collected; gender and age of household members will be recorded but no additional identifiable information will be collected. These questions will allow investigators to assess if other household members were infected or become infected with COVID-19 as a result of likely in-home transmission or community acquired infection. Daily acute COVID-19 symptom monitoring phone calls are estimated to take 10 minutes.

Study subjects will be provided with information on alarm signs and recommendations on when to seek medical attention, to assist in the event that a household member is found to be symptomatic.

3. Inpatient chart review

Patients with SpO_2 <92% or who otherwise are decompensating will be referred back to the triage centers or to a higher level. In these cases, investigators will abstract data on clinical course and patient outcomes from relevant health facilities. A standard data abstraction form will be completed for study subjects who were hospitalized or received outpatient supplemental oxygen in the COVID screening location. Data

collected will include patients' disposition at admission and discharge, treatment received, and length of stay. Each inpatient chart review is estimated to take two hours, including travel time for study staff.

4. Follow-up hand hygiene assessment

Serial follow-up WASH survey will be administered every week and at discharge (from acute COVID-19 daily monitoring) during the daily follow-up phone calls for the HBC study. The follow-up hand hygiene assessment will repeat questions about participant-reported in-home hand hygiene practices. The follow-up questionnaire may also assess the safe usage of the ABHS in the home by asking if there have been any adverse events (e.g. splash in eye or ingestion). This questionnaire is estimated to take 5 minutes. Additionally, to understand behavioral and preferential aspects of different hand hygiene technologies, upon discharge from the period of acute monitoring, 10 patients from each ABHS arm will be selected to complete Hand-Hygiene In-depth Interviews which are estimated to take 5-10 minutes. All patients enrolled in the study are eligible for selection for interviews. The 10 patients will be selected as a convenience sample based on availability of study staff.

5. Exit interview from acute COVID-19 monitoring

All study participants will be administered an exit interview to understand their opinions and level of satisfaction with the care provided in each study arm. This questionnaire is estimated to take 5 minutes.

6. Acceptability assessment

Questionnaires will be administered to study staff, triage center staff, and other key stakeholders to assess acceptability of HBC strategies. These questionnaires will explore the acceptability of the two HBC methods in providing care to patients with COVID-19 who are discharged home. A short version of the questionnaire will be used with each category of key respondent, with data stored on a secure database. Then, a longer version of the questionnaire will be administered by study staff during in-depth interviews to further explore any acceptability-related questions and suggestions and to validate initial findings. Better understanding the acceptability of the intervention and how it could be improved will allow better tailoring for larger-scale implementation. The short version of the questionnaire is estimated to take ten minutes, the in-depth interviews are estimated to take 45 minutes.

7. Qualitative WASH assessment

Questionnaires will be administered to a small group of patients who did and not receive additional ABHS in their COVID Care Kits to assess change in knowledge, attitudes, and behavior related to hand hygiene among patients and their family members during the course of their COVID-19 infection. The questionnaire will take up to 25 minutes and will be conducted over the phone by trained study personnel. All data will be securely stored.

8. Chronic COVID-19 symptoms

Following discharge from acute COVID-19 daily monitoring, study staff will offer all study participants the opportunity to participate in ongoing COVID-19 symptom monitoring. Study staff may administer phone surveys to study participants every 3 months for up to one year to assess COVID-19 sequelae and use of health services. Participants may be offered a medical assessment during the follow-up period of symptoms monitoring. Data will be collected using a standardized Post-COVID Conditions Questionnaire. Longitudinal follow-up phone surveys to assess for chronic COVID-19 symptoms are estimated to take 5 minutes.

Indicators

Specific indicators will be used to assess the feasibility, impact and acceptance of the HBC intervention. Primary outcome indicators are designated with "*". Although final indicators may be updated and/or modified, the preliminary set of indicators are listed below.

- Feasibility
    - Proportion that receive the prescribed HBC intervention
    - Proportion with complete pulse oximetry data, for relevant arm
    - Proportion lost to follow-up

- Referral to higher level of care
    - Proportion referred to higher level care due to hypoxia (i.e. SpO2 < 92%)
    - Proportion referred to higher level care due to alarm symptoms
    - Proportion referred to higher level care for any other reason
    - Proportion referred that reach higher level of care within 24 hours
    - Proportion who successfully complete HBC without requiring referral
    - Disposition after referral (home, admission, other) *
    - SpO2 level upon arrival to higher level of care
    - Other clinical measures upon arrival to higher level of care

- Impact on patient outcomes:
    - Duration of hospitalization (days)*
    - Duration of ICU and/or ventilator requirement*
    - Mortality*

- Acceptability
    - Perception of utility/benefit of strategy (study staff/patients)
    - Perception of ease of implementation of strategy (study staff)
    - Changes in hand hygiene behavior

Data Management

Data collection instruments

Study data will be entered directly into specialized data collection software on electronic tablets by study staff, with a unique identifier automatically generated at the time of subject enrollment. A unique electronic form will be created for each questionnaire listed above. The key for linking records will be stored electronically in a password protected database. See "Data Security" for additional details. On a daily basis, databases will automatically be backed up to a second secured server at a different geographic location than the primary project server.

Data monitoring dashboard

A dashboard will track and visualize key quality control metrics in real time that will include (i) facility specific enrollment, (ii) patient follow-up measures, (iii) questionnaire completeness, (iv) chronic COVID-19 symptom monitoring, (v) withdrawal and loss to follow-up. By monitoring the collection and processing of data in real time, we will be able to quickly identify and address issues that may arise during the project.

Sample size for primary outcome indicators

To calculate the power to detect a difference between two specific groups, we used a two-sided test of independent proportions with the statistical parameters of alpha 0.05, beta 0.2, and power of 0.8. The intervention effect was selected to provide clinically and operationally meaningful outcomes. In addition, the power calculations are based on the following assumptions.

- Likelihood of referral for higher care: According to a study on the use of pulse oximeters to determine referral of COVID-19 patients to a higher level of care, 24.7% of patients required such a referral, in a second study 33% of patients using a pulse oximeter at home and receiving remote COVID-19 management were referred.[7,13] Given that those studies were conducted prior to the availability of vaccines, and 34% of the Honduran population is vaccinated, we will use a baseline estimate that 20% of patients will be referred for higher care, with a greater proportion referred in the group using pulse oximeters, given additional opportunities to detect the need for referral.[5] The ability to detect a 6% absolute difference between referral rates between arms requires an enrollment of 804 patients per arm for a total enrollment of 1,608 participants.

- Likelihood of hospitalization: The most recent published COVID-19 study in Honduras identified a 25% hospitalization rate in the general population, prior to vaccine availability.[14] We estimate that 10% of all enrolled patients will be hospitalized, taking into account increasing vaccination coverage and the high risk status of the enrolled population. The ability to detect a 5% absolute difference in hospitalization rates between arms requires 946 participants per arm for a total enrollment of 1,892 participants.

- Length of hospitalization: Given an average of 7.7 days hospitalization per admitted patient (SD 10.8), a per arm/strategy sample size of 773 is powered to detect a 20% difference in length of hospitalization between groups for a total enrollment of 1546 participants.

- Pulse oximetry: Given an estimated mean SpO2 of 93% (SD 7%) at the time of evaluation at a higher level of care, a per arm/strategy sample size of 85 is powered to detect an absolute difference between groups of SpO2 of 3%, for a total of 255 patients referred to a higher level of care, for a total enrollment of 850 participants.

- Mortality: Given an estimated mean mortality of 7.5% among the enrolled population, a per arm sample size of 854, and a total sample of 1708, is powered to detect a difference in mortality rate of 3.3% between groups. The mean mortality rate among people with COVID in Honduras is 2.8% among the general population.[5] Given that the enrolled population will be older and more likely to live with comorbidities for negative COVID outcomes, a higher estimated mortality rate has been used.

Covariate	General population (SD)
Proportion of COVID-19 patients requiring hospitalization	25%
Duration of hospitalization[15]	7.7 days (10.8)
SpO2 at time of evaluation at higher level of care	93% (7%)

Data analysis

The primary analysis will provide unadjusted and adjusted differences between arms/strategies based on the covariates listed. We will perform additional subgroups analyses, specifically examining age, sex, comorbidities, baseline clinical features, and timing of the interventions. Qualitative analyses will be conducted by trained researchers using Excel to organize data, given the limited sample sizes. Research analysis will adhere to the Sex and Gender Equity in Research (SAGER) guidelines including reporting representativeness, presenting disaggregated results regardless of positive or negative outcome, withdrawal/attrition rates, and implications of reported gender on study results.

Training and Qualifications of Personnel

Study staff will be trained to enter study data directly into electronic tablets. Study staff will be trained in enrollment and consent procedures, administration of study entry questionnaires, providing home-based care and how to obtain information about COVID-19 outcomes from patients, their family members and/or facilities to which they are referred for high levels of care. They will also be trained in research ethics and database management.

Risks and Benefits to Participants

Risks to confidentiality
There is a low risk that study databases could be accessed, and patient information obtained. However, all data maintained in the database will be de-identified with only the PI, project manager, and data manager able to access the key to de-identify data. The patient monitoring form does not include sensitive questions that would be expected to be stigmatizing or cause emotional distress. Further, we will ensure participants know that they do not have to answer any questions that make them uncomfortable. Only study personnel will be allowed to review this information. All data will be entered directly into the electronic database and no paper records will be maintained. We will adhere to best practice mechanisms to ensure data security as described below. Evaluation staff will receive training in confidentiality and the protection of personally identifiable information.

Risk of exposure to study staff
There is a low risk that study staff could potentially be infected while interacting with study participants or could potentially be infectious and transmit to study participants. Study staff will follow all infection prevention guidelines, including mask-wearing, hand hygiene, and social distancing. Any staff traveling between Honduras and the United States will be required to be vaccinated against SARS-CoV-2.

Reporting of potential adverse events
Given the nature of the study, the risk of adverse events related to study participants is low. However, the project will monitor for unexpected or unintended adverse events related to patient safety by ensuring all project staff are trained on the identification and reporting of these events. Reporting will be to the Project Manager and these will be reported at a minimum monthly, or more frequently as PI requests, to the PI. Serious adverse events will also be reported to the respective Honduras, CDC, and Mass General Brigham IRB committees.

Benefits

Individual participants will benefit by receiving an HBC kit. They will also receive follow-up from a medical worker which could help to more rapidly identify decompensation and facilitate referral to a higher level of care when necessary, which could lead to improved outcomes. There will also be societal benefits. Results will be shared with policymakers at the Ministry of Health and COVID-19 Taskforce, which can be used to inform and improve policies and programs for COVID-19 HBC and for HBC in general.

Data security

Survey data will be collected using KoBoToolbox, a widely used cloud-based data collection and management platform on electronic tablets. Data will be stored on the tablets and automatically uploaded into a password-secured cloud-based database when internet access is available. Data will be hosted on Amazon Web Services server, administered using best practice tools and mechanisms to ensure data security and prevent loss. Data will be exported in CSV format for analysis by the research team, although users can also use the Excel Data Analyzer developed by UN OCHA to support preliminary analyses. Access permission will only be granted by a study PI. At least two backups of the two generations of database tables will be maintained. An automatic backup will be included in the system while invoking the module for detecting keypunch errors. Potentially identifiable data shared with the health ministry will be performed with secure file transfer. A data security risk assessment will be performed by the Mass General Brigham Healthcare Information Security and Privacy Office to ensure data security and integrity and all project electronic tablets and computers used for data entry and storage will be encrypted using the MobileIron mobile security platform (www.mobileiron.com/en/security-and-compliance).

Human subjects' considerations

Informed consent

Written informed consent will be obtained from all study participants via electronic signature collection on a tablet (Appendices 1 and 2). Consent will be obtained by study staff trained by the research team in obtaining informed consent. If the patient is illiterate, the relevant informed consent form will be verbally read to them by staff in Spanish. The participant, minor, parent, or guardian will be requested to use an X on the tablet screen using their finger in lieu of a signature in the presence of witness who is not involved in this project. The surveillance staff member will fill out and sign the form documenting the completion of consent procedures and, if the patient meets the eligibility criteria, they will be enrolled. For qualitative data collection, written consent will be obtained if conducted in person, whereas verbal consent will be obtained if conducted over the phone.

Vulnerable subjects

Pregnant women will be enrolled, but not specifically identified for enrollment and there is no increased risk to pregnant women. The data and outputs of this study are relevant for pregnant women, particularly given that pregnant women are a high-risk group for complications of COVID-19. Prisoners will not be enrolled in this study.

Data sharing and dissemination policies

All investigators are aware of and agree to abide by the principles for sharing research resources, as described by NIH in "Principles and Guidelines for Recipients of NIH Research Grants and Contracts on Obtaining and Disseminating Biomedical Research Programs." A data governance document will be

developed to define the rights each investigating institution has with regards to data, the roles and responsibilities of partners for data governance and to define the timeline and mechanism for certifying finalization of data at the end of the survey and releasing datasets. In keeping with overall goal ensuring the data and outputs from the project achieve maximal public health impact the data sharing plan is as follows:

1. SESAL is the owner of the data and will have access to the data and necessary tools for accessing the data both during data collection and after closure of the project. The BWH Project Team together with the CDC has responsibility for data management and analysis. Any scientific publications or presentations developed as a result of such analyses will be done in collaboration between SESAL, CDC, and BWH Project Team. No results will be published without the approval of SESAL.
2. The BWH Project Team as part of the Cooperative Agreement with the US CDC will share de-identified study data with CDC. CDC staff will have access to only de-identified study data to review study data for quality and will conduct any data analysis in collaboration with SESAL and the BWH Project Team.
3. The BWH Project Team will lead the analyses of the evaluation, and, in collaboration with CDC and SESAL, produce priority results in table form for sharing with SESAL and other key stakeholders in-country, as well as a formal report before any abstracts or manuscripts are developed. Priority results will be disseminated within six weeks of the end of data collection.
4. The BWH Project Team, CDC, and SESAL will prepare abstracts on the findings for presentation at research meetings. We particularly will concentrate our efforts to present the products of this work in Honduras at relevant conferences and special meetings. In addition, we plan to present our work at international conferences that are particularly relevant to the Caribbean and Central America.
5. The BWH Project Team, CDC, and SESAL will prepare research findings for publication in peer-reviewed journals. SESAL approval is required for publication of any data related to this project. Individuals from the three collaborating institutions (BWH, CDC, and SESAL) will be included as authors on all publications. Whenever possible, we aim to publish in open access journals so that the work can be readily available to our target audience in countries in the Caribbean and Central America. All peer-reviewed manuscripts that arise from this proposal will be submitted to the digital archive PubMed Central.

Non-identifiable data required to reproduce study results will be made publicly available alongside each publication. Data will be stored as a machine-readable CSV file and published open access under a CC-BY license.

References

1. **Home care for patients with suspected or confirmed COVID-19 and management of their contacts.** https://www.who.int/publications-detail-redirect/home-care-for-patients-with-suspected-novel-coronavirus-(ncov)-infection-presenting-with-mild-symptoms-and-management-of-contacts.
2. Secretaria de Salud de Honduras (SESAL). *Guía Práctica Para El Manejo Domiciliar De Pacientes Sospechosos o Confirmados por COVID-19*. http://www.salud.gob.hn/site/index.php/component/edocman/24-02-21-version-final-sesal-guia-practica-para-el-manejo-domiciliar-de-pacientes-sospechosos-o-confirmados-por-covid-19 (2021).
3. World Health Organization. Coronavirus Disease (COVID-19). https://www.who.int/health-topics/coronavirus?fbclid=IwAR03e7bgDgivYWTEmdWyWW8mOFWxYMbynaOE3eVTeO7_meV8rMMIbz9FdC0#tab=tab_1.
4. Impacts of COVID-19 disproportionately affect poor and vulnerable: UN chief. *UN News* https://news.un.org/en/story/2020/06/1067502 (2020).
5. Geo-Hub COVID-19 - Information System for the Region of the Americas. https://paho-covid19-response-who.hub.arcgis.com/.
6. Jouffroy, R., Jost, D. & Prunet, B. Prehospital pulse oximetry: a red flag for early detection of silent hypoxemia in COVID-19 patients. *Crit. Care* **24**, 313 (2020).
7. Shah, S. *et al.* Novel Use of Home Pulse Oximetry Monitoring in COVID-19 Patients Discharged From the Emergency Department Identifies Need for Hospitalization. *Acad. Emerg. Med.* **27**, 681–692 (2020).
8. Xie, J. *et al.* Critical care crisis and some recommendations during the COVID-19 epidemic in China. *Intensive Care Med.* **46**, 837–840 (2020).
9. Edejer, T. T.-T. *et al.* Projected health-care resource needs for an effective response to COVID-19 in 73 low-income and middle-income countries: a modelling study. *Lancet Glob. Health* **8**, e1372–e1379 (2020).
10. Rijal, S. & Poudel, B. Is home pulse oximeter monitoring for COVID-19 feasible in low-income and low-middle-income countries? *BMJ Health Care Inform.* **28**, e100465 (2021).
11. Gootenberg, D. B. *et al.* Developing a pulse oximetry home monitoring protocol for patients suspected with COVID-19 after emergency department discharge. *BMJ Health Care Inform.* **28**, e100330 (2021).
12. Advice for the public on COVID-19 – World Health Organization. https://www.who.int/emergencies/diseases/novel-coronavirus-2019/advice-for-public.
13. Greenhalgh, T. *et al.* Remote management of covid-19 using home pulse oximetry and virtual ward support. *BMJ* **372**, n677 (2021).
14. Zuniga-Moya, J. C. *et al.* Epidemiology, Outcomes, and Associated Factors of Coronavirus Disease 2019 (COVID-19) Reverse Transcriptase Polymerase Chain Reaction–Confirmed Cases in the San Pedro Sula Metropolitan Area, Honduras. *Clin. Infect. Dis.* **72**, e476–e483 (2021).
15. Risk Factors Associated With In-Hospital Mortality in a US National Sample of Patients With COVID-19 | Cardiology | JAMA Network Open | JAMA Network. https://jamanetwork.com/journals/jamanetworkopen/fullarticle/2773971.
